# Individual Organ Failure and Concomitant Risk of Mortality Differs According to the Type of Admission to ICU – A Retrospective Study of SOFA Score of 23,795 Patients

**DOI:** 10.1371/journal.pone.0134329

**Published:** 2015-08-04

**Authors:** Tobias M. Bingold, Rolf Lefering, Kai Zacharowski, Patrick Meybohm, Christian Waydhas, Peter Rosenberger, Bertram Scheller, DIVI Intensive Care Registry Group

**Affiliations:** 1 Department of Anesthesiology, Intensive Care Medicine and Pain Therapy, University Hospital Frankfurt am Main, Theodor-Stern-Kai 7, D-60590 Frankfurt am Main, Germany; 2 Institute for Research in Operative Medicine (IFOM), University of Witten/Herdecke, Cologne-Merheim Medical Center (CMMC), Ostmerheimerstrasse 200, D-51109 Cologne, Germany; 3 Trauma Surgery Department, University Hospital Essen, Hufelandstrasse 55, D-45122 Essen, Germany; 4 Department of Anesthesiology and Intensive Care Medicine, Eberhard Karls Universität, D-72076 Tübingen, Germany; Imperial College London, Chelsea & Westminster Hospital, UNITED KINGDOM

## Abstract

**Introduction:**

Organ dysfunction or failure after the first days of ICU treatment and subsequent mortality with respect to the type of intensive care unit (ICU) admission is poorly elucidated. Therefore we analyzed the association of ICU mortality and admission for medical (M), scheduled surgery (ScS) or unscheduled surgery (US) patients mirrored by the occurrence of organ dysfunction/failure (OD/OF) after the first 72h of ICU stay.

**Methods:**

For this retrospective cohort study (23,795 patients; DIVI registry; German Interdisciplinary Association for Intensive Care Medicine (DIVI)) organ dysfunction or failure were derived from the Sequential Organ Failure Assessment (SOFA) score (excluding the Glasgow Coma Scale). SOFA scores were collected on admission to ICU and 72h later. For patients with a length of stay of at least five days, a multivariate analysis was performed for individual OD/OF on day three.

**Results:**

M patients had the lowest prevalence of cardiovascular failure (M 31%; ScS 35%; US 38%), and the highest prevalence of respiratory (M 24%; ScS 13%; US 17%) and renal failure (M 10%; ScS 6%; US 7%). Risk of death was highest for M- and ScS-patients in those with respiratory failure (OR; M 2.4; ScS 2.4; US 1.4) and for surgical patients with renal failure (OR; M 1.7; ScS 2.7; US 2.4).

**Conclusion:**

The dynamic evolution of OD/OF within 72h after ICU admission and mortality differed between patients depending on their types of admission. This has to be considered to exclude a systematic bias during multi-center trials.

## Introduction

Scoring systems for the prediction of mortality in ICU patients often incorporate three main categories for “type of admission”, namely “scheduled surgery”, “unscheduled surgery” and “medical” [1–3]. Another important scoring system objectively describes the degree of organ dysfunction and/or failure, and their associated morbidity over time (Sequential Organ Failure Assessment (SOFA) Score) [4]. A retrospective analysis primarily designed to assess morbidity using the European/North American Study of Severity System database found a close correlation between the SOFA score at ICU admission (SOFA_adm_) and patient outcome [4]. This finding was further validated by a subsequent prospective observational study that evaluated data from 1,449 critically ill patients treated in 40 ICUs.[5] Mean and highest SOFA scores measured over the first few days following ICU admission were strong prognostic indicators. Independent of the patient’s initial score on ICU admission and irrespective of type of admission, an increase in SOFA score over the first 48 hours predicted a mortality rate of at least 50% [6].

The individual organ failure of patients mainly determines the individual risk of death. After an episode of critical illness in Scottish ICUs, cardiovascular failure (odds ratio [OR], 2.5; 95% confidence interval [CI], 1.8–3.7), liver failure (OR, 2.3; 95% CI, 1.1–5.0) and respiratory failure (OR, 2.1; 95% CI, 1.3–3.5) were independently associated with 5-year mortality [7]. The impact of the type of ICU admission on individual organ failure and subsequent mortality however remains poorly elucidated. We therefore examined this question in a large multicenter German database, focusing on patients with a longer ICU stay. Using multivariate regression analysis we tested whether a specific organ dysfunction or failure presenting within 72 hours after ICU admission maps to the same risk of death in patients admitted irrespective of their type of admission. Vincent et al. demonstrated in a retrospective evaluation of activated Protein C database (INDEPTH), that SOFA score increased in the days before death [8]. As rapidly deteriorating multi organ dysfunction may introduce a bias, this multivariate analysis was restricted to patients with an ICU length of stay of at least five days.

## Materials and Methods

**Fig 1:**
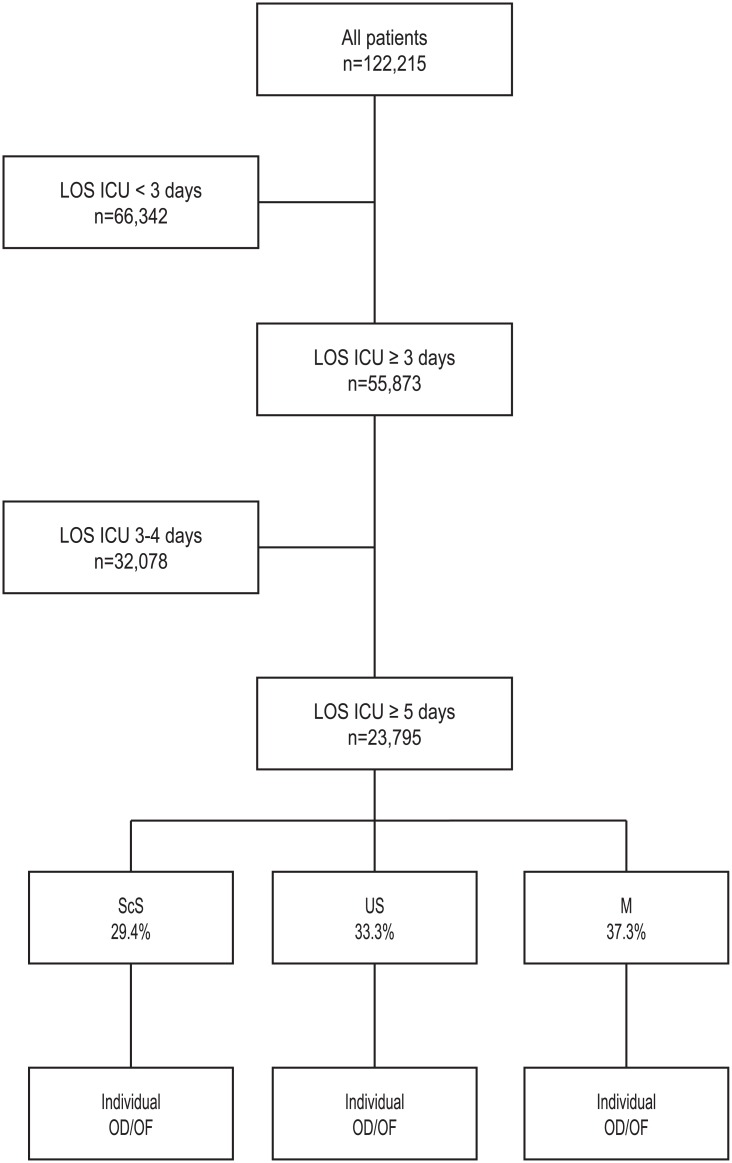
Study protocol. ICU length of stay ≥ 5 days, classifying patients according to the type of admission (percent of patients), followed by an evaluation of individual organ dysfunction/organ failure on ICU day three. ScS, scheduled surgery; US, unscheduled surgery; M, medical; SOFA; OD, organ dysfunction (SOFA score 1–2 points); OF organ failure (SOFA score 3–4 points).

This study represents a retrospective analysis of a registry including 122,215 patients treated at 75 ICUs from 2000 to 2010, and prospectively documented in the German Interdisciplinary Association for Intensive Care Medicine (DIVI) registry (Fig 1). The study was approved by the local ethics committee of the University Hospital, Frankfurt. Patient records/information and hospital records/information were anonymized and de-identified prior to analysis. According to the ethics committee individual patient consent was not required.

### DIVI registry

The interdisciplinary registry contains a core dataset for intensive care medicine in Germany [9]. It contains data regarding the type and structure of the participating ICUs (recorded annually), admission (recorded once per patient) and daily patient data (SAPS II, SOFA, and the Simplified Therapeutic Intervention Scoring System (TISS)-28), and ICU discharge data [9, 10]. The database is organized and funded by DIVI.

### The types of hospitals

The registry included ICU patients from a broad spectrum of hospitals including small community hospitals, university hospitals and specialized care hospitals, irrespective of their medical disciplines (Table 1). The level of care (LOC) of patients admitted to the ICUs was mainly LOC 2–3 [11].

**Table 1:** The departments to which the patients were admitted (%).

	% of patients
**General Surgery**	25
**Internal Medicine**	22
**Cardiothoracic Surgery**	11
**Neurosurgery**	10
**Traumatology**	9
**Urology**	4
**Vascular Surgery**	4
**Gynecology**	3
**Orthopedics**	3
**Neurology**	2
**Others**	7

### Inclusion criteria

- ICU length of stay (LOS) of at least five days
- SOFA scores on admission and day three available
- Type of ICU admission available
- ICU discharge status (mortality) available
- Age and sex available

### SOFA score

The SOFA score describes organ function of respiratory, cardiovascular, renal, hematologic, hepatic, and central nervous systems [4]. The DIVI database excluded the Glasgow Coma Scale (GCS) from the SOFA score because of the difficulty in assessment in sedated and/or paralyzed patients.

Each organ system is graded using a five-step scale (0–4 points) to evaluate the severity of organ dysfunction/failure. Specifically, 1–2 points (per organ) are awarded for ‘dysfunction’ and 3–4 points (per organ) are granted for ‘failure’.

### Specific organ dysfunction/organ failure and risk of death

Specific organ dysfunction or failure was evaluated on day three following admission to ICU. Since rapidly deteriorating MODs may introduce a bias when approaching death, we restricted this analysis to a dataset of 23,795 patients with an ICU length of stay of at least five days (Fig 1).

The prevalence of each organ dysfunction (SOFA score 1–2 points) and failure (SOFA score 3–4 points) was evaluated. In addition, a multivariate logistic regression analysis was performed, evaluating the odds ratio with ICU mortality as the dependent variable for each organ system, adjusted for type of ICU admission, age and gender. A second multivariate regression analysis was performed for each type of admission, adjusted for age and gender, with respect to the individual organ dysfunction or failure measured on day three of ICU admission.

### Statistics

Continuous variables are presented as mean and standard deviation (SD). Categorical variables are presented as counts and percentages.

The main outcome criterion was ICU mortality. Mortality rates were determined for specific patient subgroups according to SOFA_adm_ and SOFA_72h_. Additional subgroups were formed according to the type of ICU admission.

The risk of mortality for the different types of failed organ systems was assessed by multivariate logistic regression analysis, with ICU mortality as the dependent variable. Adjustments were made for age, gender and type of admission. The adjusted impacts of different types of organ dysfunction or failure are presented as odds ratios (OR) with 95% confidence intervals (CI).

Formal statistical testing was avoided due to the large number of cases, which would result in statistically significant *p*-values even for minor and clinically irrelevant differences. All of the analyses were performed using the SPSS statistical software package (version 20.0, IBM, Inc., Armonk, NY, USA).

## Results

### Baseline characteristics

In total, data were collected from 122,215 patients admitted to 75 ICUs (Fig 1). Mean patient age was 63.4 ± 17.0 years, and mean ICU length of stay was 4.2±7.8 days.

A total of 23,795 patients met the inclusion criteria of an ICU length of stay of at least five days (Fig 1; Tables 2 and 3).

**Table 2:** Characteristics of the patients with an ICU LOS of at least 5 days.

		All patients	Survivors	Non-survivors
**Patients, No. (%)**		23,795	20,196 (84.9)	3,599 (15.1)
**LOS ICU, mean ±sd**		13.57±13.4	12.79±12.4	17.9±17.5
**Age years, mean±sd**		64.2±16.5	63.31±16.8	68.8±13.6
**Gender**				
	**Male No. (%)**	13,904 (56.0)	11,793 (58.4)	2,111 (58.7)
	**Female No. (%)**	9,891 (44.0)	8,403 (41.6)	1,488 (41.3)
**SAPS II, mean±sd**		31.3±13.3	29.8±12.6	39.9±14.0
**SOFA**				
	**SOFA**_**adm**_, **mean±sd**	4.53±3.1	4.25±3.0	6.11±3.4
	**SOFA**_**72h**_, **mean±sd**	4.65±3.2	4.27±3.0	6.78±3.4
**LOS ICU**				
	**Days, mean±sd**	13.6±13.4	12.8±12.4	17.9±17.5
**Ventilation**				
	**Ventilated, No. (%)**	18,959 (79.7)	15,531 (76.9)	3,428 (95.2)
**Type of admission**				
	**Medical, No. (%)**	8,873 (37.3)	7,273 (36.0)	1,600 (44.4)
	**Scheduled surgery, No. (%)**	6,983 (29.4)	6,198 (30.7)	785 (21.8)
	**Unscheduled surgery, No. (%)**	7,939 (33.3)	6,725 (33.3)	1,214 (33.4)

LOS, Length of Stay; SAPS II, Simplified Acute Physiology Score II; SOFA, Sequential Organ Failure Assessment Score; SOFA_adm_, SOFA score at admission to the ICU; SOFA_72h_, SOFA score at 72 h; SOFA_delta72h_, SOFA_adm_ - SOFA_72h_

**Table 3:** Characteristics of the patients with an ICU LOS of at least 5 days, type of admission to ICU.

		Medical	Unscheduled Surgery	Scheduled Surgery
**Patients, No. (%)**		8,873 (37.3)	7,939 (33.3)	6,983 (29.4)
**Non survivors, No. (%)**		1600 (18.0)	1214 (15.3)	785 (11.2)
**LOS ICU, mean ±sd**		13.7±12.8	14.5±13.9	12.4±13.5
**Age years, mean±sd**		64.6±16.3	64.1±16.4	69.3±13.8
**Gender**				
	**Male No. (%)**	5,332 (56.0)	4,575 (57.6)	3,997 (57.2)
	**Female No. (%)**	3,541 (40.0)	3,364 (39.9)	2,986 (42.8)
**SAPS II, mean±sd**		34.1±13.7	33.3±13.2	25.7±10.9
**SOFA**				
	**SOFA**_**adm**_, **mean±sd**	4.59±3.2	4.67±3.1	4.29±2.9
	**SOFA**_**72h**_, **mean±sd**	4.62±3.2	4.76±3.1	4.55±3.1
**Admission**				
	**Out of hospital, No. (%)**	1,484 (16.7)	692 (8.7)	253 (3.6)
	**Emergency, No. (%)**	3,777 (42.6)	7,939 (100)	0 (0)
**Ventilation**				
	**Ventilated, No. (%)**	6470 (72.9)	6871 (86.5)	5618 (80.5)

Patients characteristics in respect to their type of admission to ICU, n = 23,795

LOS Length of Stay; SAPS II Simplified Acute Physiology Score II; SOFA, Sequential Organ Failure Assessment Score; SOFA_adm_ SOFA at admission to ICU; SOFA_72h_ SOFA at 72h

29.4% of patients were admitted after ScS, 33.3% after US, and 37.3% for a medical reason (M) (Table 2). ICU mortality rates were 18.0% (CI 95% 17.15–18.91) for M and 15.3% (14.44–16.15) for US and lower for ScS (11.2% (10.46–12.02) (Table 3).

### Prevalence of organ failure

**Fig 2:**
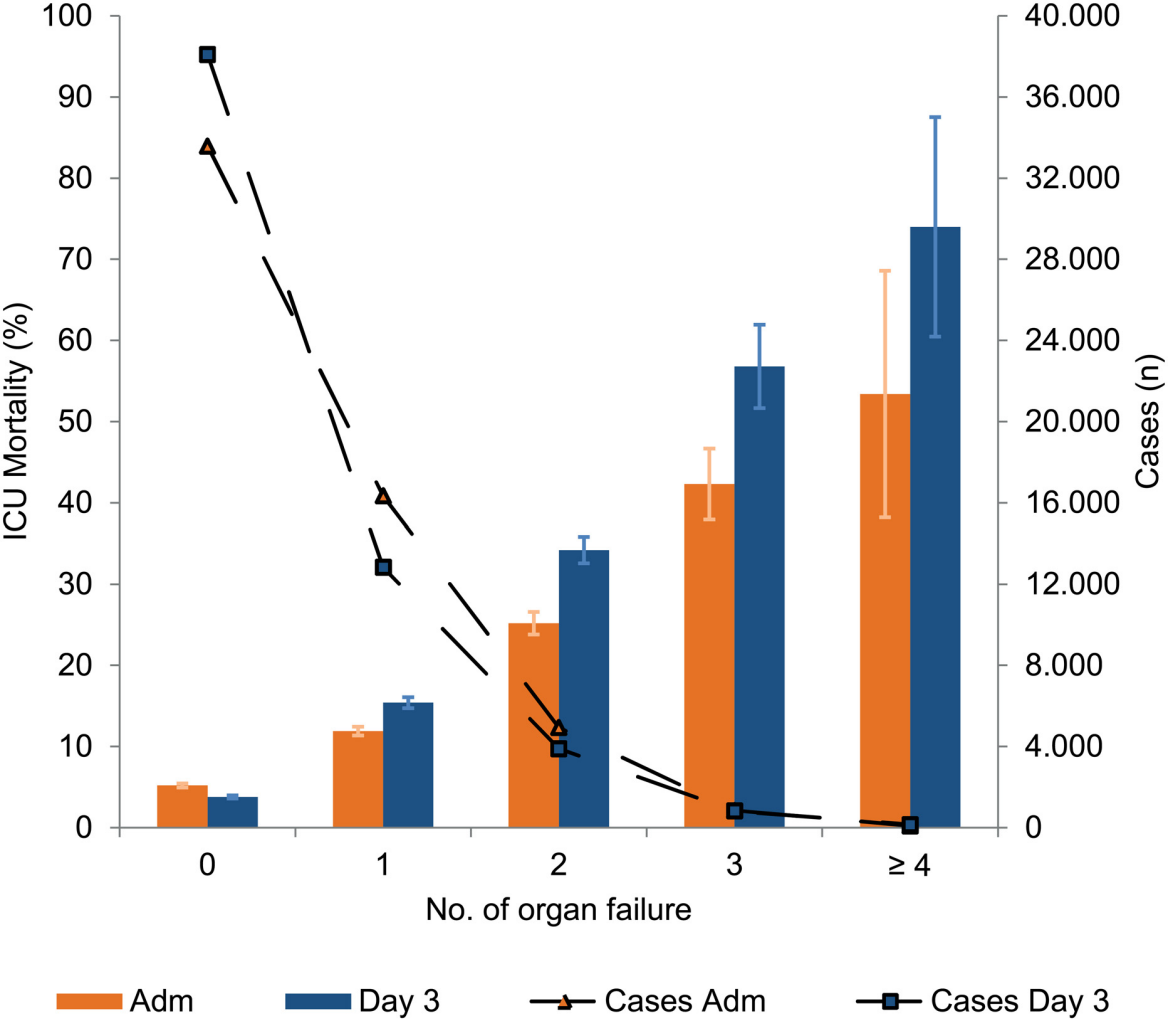
Number of organ failure on admission and day 3. Number of patients with n organ failure ad admission (rectangle); Number of patients with n organ failure on day 3 (triangle); ICU Mortality (mean group ± 95% CI) in respect to number of organ failure for 23,795 cases with an ICU length of stay of at least five days.

**Fig 3:**
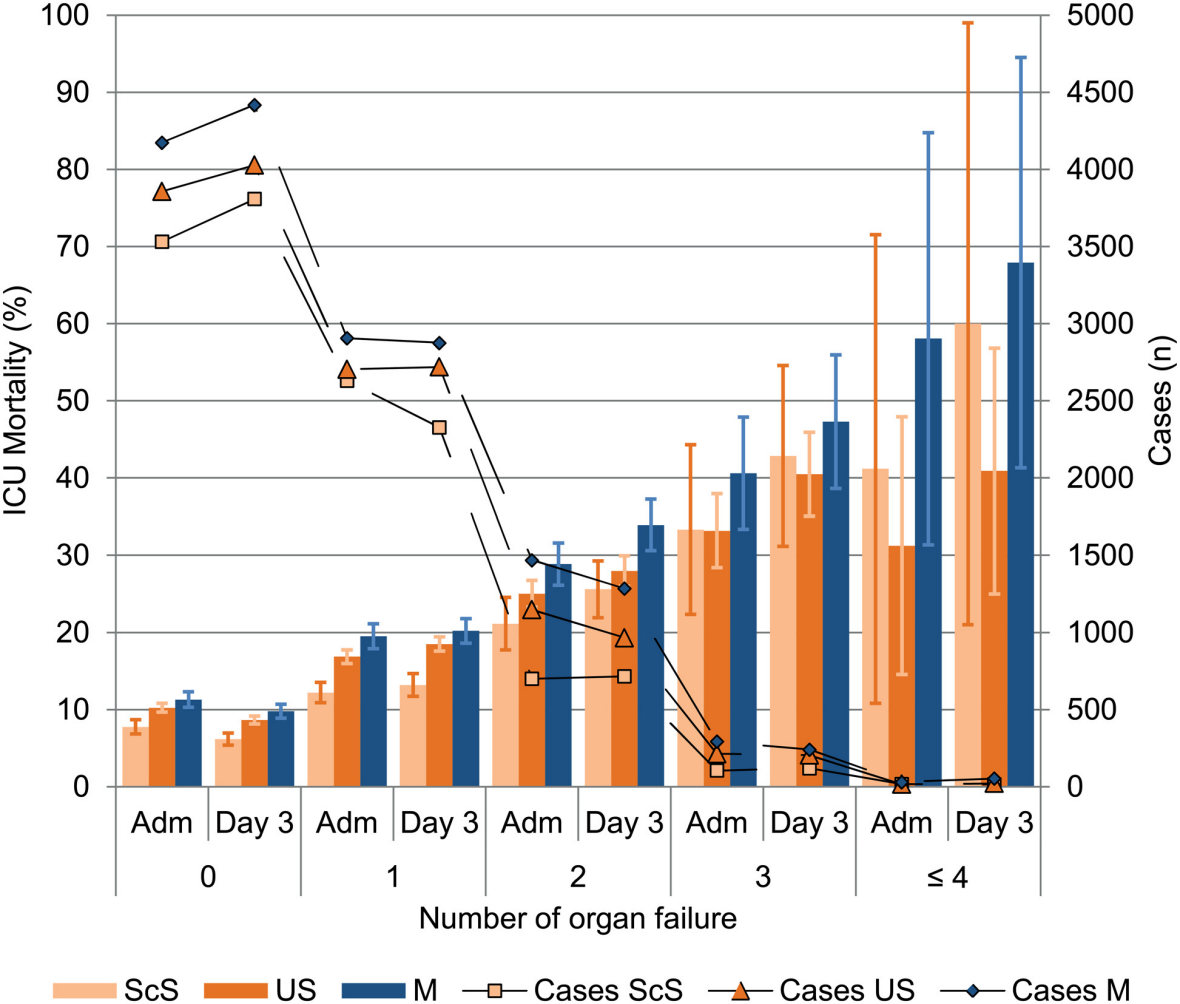
Organ failure on admission and day three of ICU treatment in the ScS-, the US- and the M-patients. Bars depict the ICU mortality rate of each organ failure (group mean ± 95% CI). Mortality increased with an increasing number of organ failures while the same number of organ failures on day three had a higher mortality rate compared to the day of ICU admission for every type of admission. M patients had higher mortality rates than surgical admitted patients. Lines depict the number of cases with an organ failure. ScS, scheduled surgery; US, unscheduled surgery; M, medical patients; Adm, ICU Admission day; day 3, third day of ICU treatment.

The prevalence of one or more organ failures in patients with an ICU length of stay of at least five days was 51.4% (Fig 2). The prevalence of one organ failure *on admission* was 34.6% overall, with the highest rate (37.7%) seen in patients after scheduled surgery (Fig 3). By contrast, US and M patients had a greater prevalence of at least two OF. After three days of ICU treatment, the rate of patients without organ failure increased to 51.5% (Fig 2). The prevalence of at least two OF was more or less unchanged between ICU admission and day three, irrespective of the type of admission.

### Mortality with respect to the number of organ failures

ICU mortality increased with an increasing number of organ failures (Fig 2). The same number of organ failures on day three as on ICU admission led to an increased mortality rate, irrespective of the type of admission. M patients had higher mortality rates on ICU admission and on day three for the same number of organ failures (Fig 3).

### The individual organ systems

**Fig 4:**
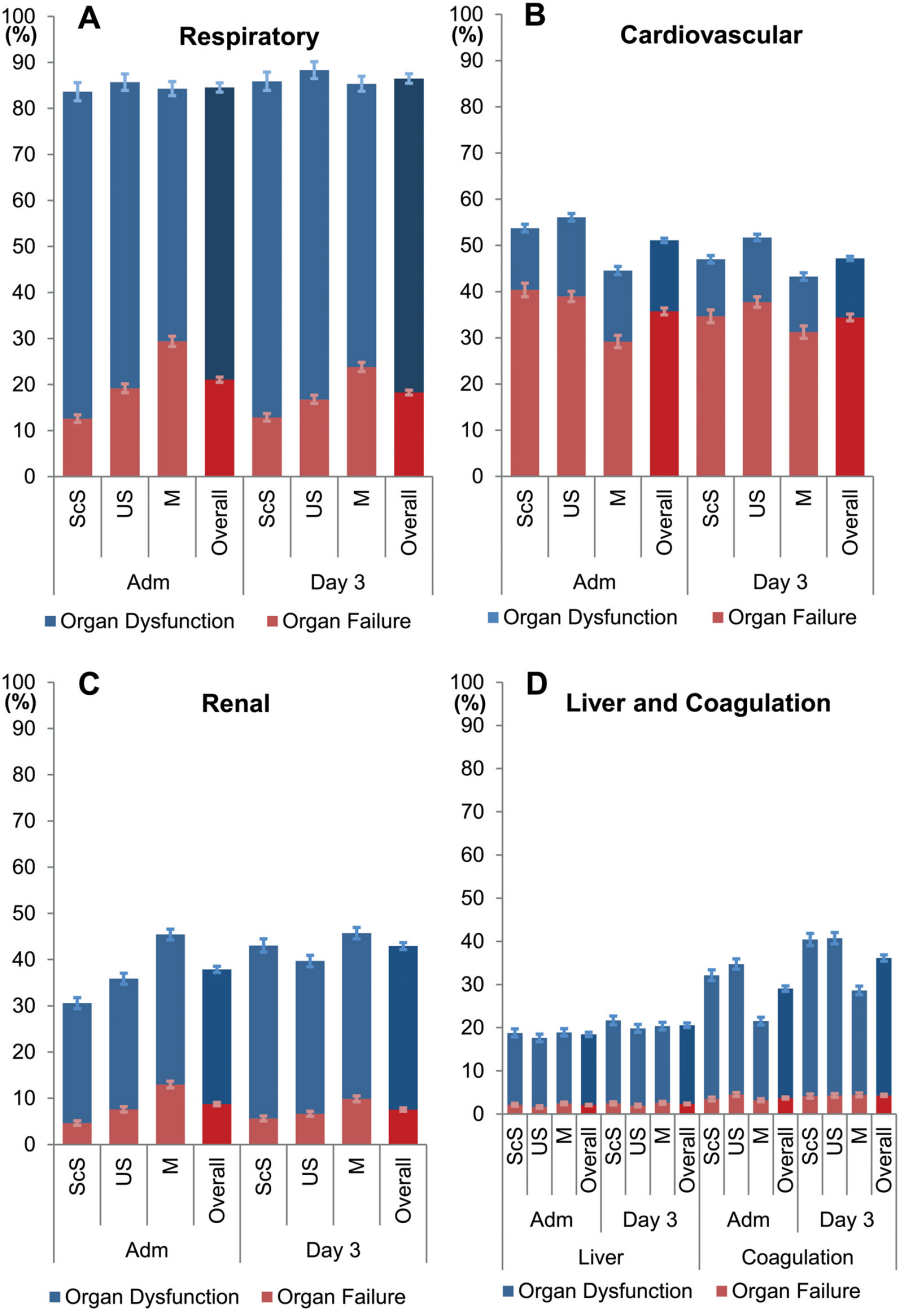
Prevalence of Organ dysfunction and failure per organ at admission to ICU and at day 3. Depicted are the prevalences (mean group ± 95% CI) of organ dysfunction (SOFA score 1–2 points) and organ failure (SOFA score 3–4 points) on admission and on day 3 of ICU in respect to the type of admission in patients with an ICU LOS of at least five days. M-patients had on admission to ICU and on day 3 less heart failure (B) and less coagulation dysfunction (D), but a higher proportion of respiratory (A) and renal failure (C) than the ScS and the US patients. ScS, scheduled surgery; US, unscheduled surgery; M, medical; Adm, admission to ICU; day 3 day 3 of ICU treatment; normal, individual organ SOFA score 0 points; dysfunction, individual organ SOFA score 1–2 points; failure, individual organ SOFA score 3–4 points.

The most common organ failure was cardiovascular both on ICU admission (35.7%) and after 72h (34.4%). However, this particular organ failure was lowest in the M patients in comparison to ScS and US patients (Fig 4).

The most frequent organ dysfunction/failure was respiratory, affecting 84.6% of patients on ICU admission and 86.5% on day three. M patients had a higher rate of respiratory failure compared to dysfunction (29.4% versus 54.9%, respectively) in comparison to ScS (12.6% vs 71.1%) or US (19.2% vs 66.5%) patients (Fig 4).

### Multivariate analysis of organ dysfunction and organ failure

*At admission to ICU* odds ratio with intensive care unit mortality as the dependent variable was nearly equal for each individual organ dysfunction (OR 1.229–1.335) except of a renal dysfunction (OR 1.408; 95% CI 1.295–1.531) (Table 4). This multivariate analysis was adjusted to gender, age and type of admission.

**Table 4:** Multivariate, forward stepwise logistic regression analysis in patients with an ICU length of stay of at least 5 days (n = 23,795), with intensive care unit mortality as the dependent factor.

		OR	95% CI	p Value
**Organ dysfunction/organ failure at admission**				
CV dysfunction		1.288	(1.152–1.440)	< .001
CV failure		1.787	(1.640–1.947)	< .001
Lung dysfunction		1.229	(1.087–1.390)	< .001
Lung failure		1.828	(1.596–2.092)	< .001
Renal dysfunction		1.408	(1.295–1.531)	< .001
Renal failure		1.873	(1.665–2.107)	< .001
Liver dysfunction		1.335	(1.216–1.466)	< .001
Liver failure		2.587	(2.098–3.189)	< .001
Coagulation dysfunction		1.292	(1.187–1.407)	< .001
Coagulation failure		1.690	(1.423–2.007)	< .001
**Organ dysfunction/organ failure admission and day 3**				
CV dysfunction	ADM	.923	(.814–1.047)	.215
CV failure	ADM	.993	(.895–1.102)	.897
Lung dysfunction	ADM	1.087	(.929–1.272)	.296
Lung failure	ADM	1.220	(1.023–1.454)	.027
Renal dysfunction	ADM	1.057	(.948–1.178)	.316
Renal failure	ADM	1.146	(.971–1.353)	.107
Liver dysfunction	ADM	1.027	(.901–1.171)	.690
Liver failure	ADM	.941	(.655–1.351)	.740
Coagulation dysfunction	ADM	.959	(.857–1.074)	.468
Coagulation failure	ADM	.932	(.749–1.160)	.531
CV dysfunction	Day 3	1.939	(1.702–2.208)	< .001
CV failure	Day 3	2.545	(2.295–2.822)	< .001
Lung dysfunction	Day 3	1.090	(.918–1.295)	< .001
Lung failure	Day 3	1.765	(1.459–2.136)	.323
Renal dysfunction	Day 3	1.438	(1.290–1.602)	< .001
Renal failure	Day 3	1.888	(1.583–2.250)	< .001
Liver dysfunction	Day 3	1.349	(1.190–1.529)	< .001
Liver failure	Day 3	2.754	(1.966–3.859)	< .001
Coagulation dysfunctio	Day 3	1.379	(1.238–1.536)	< .001
Coagulation failure	Day 3	2.229	(1.824–2.725)	< .001

***Organ dysfunction/organ failure at admission*** depicts the risk of death for patients with organ dysfunction/organ failure on ICU admission, with a significant increase in the risk of death for all organ dysfunctions/organ failures.

***Organ dysfunction/organ failure at admission and day 3*** depict the risk of death for organ dysfunction/organ failure on ICU admission and on day 3. Here the impact of organ dysfunction/organ failure seen at ICU admission diminishes; except for patients admitted with respiratory organ dysfunction, all organ failure had a significant impact on the risk of death. All data are adjusted for age, gender and type of admission.

OR, odds ratio; CI, confidence interval; CV, cardiovascular; ADM, individual organ dysfunction/organ failure at admission to ICU.

In patients with at least one organ failure, the highest risk of death was observed with liver failure (OR 2.587; CI 2.098–3.189) followed by a nearly similar odds ratios for renal-, lung-, heart- and coagulation failure ranging from 1.690 to 1.873 (Table 4).

However on day three, no organ dysfunction or organ failure present on ICU admission (except for lung), once adjusted for gender, age and type of admission, had a significant influence on mortality (Table 4). An organ dysfunction present on day three had a significant impact on mortality except lung dysfunction. The highest risk of death was observed with cardiovascular dysfunction (OR 1.939; 95% CI 1.702–2.208). In patients with an OF present on day three an organ failure of the liver had the highest odds ratio (2.754; 95% CI 1.966–3.859) followed by a failure of the cardiovascular-, coagulation-, renal-system and the lungs (Table 4).

### Type of admission and organ dysfunction / organ failure

**Fig 5:**
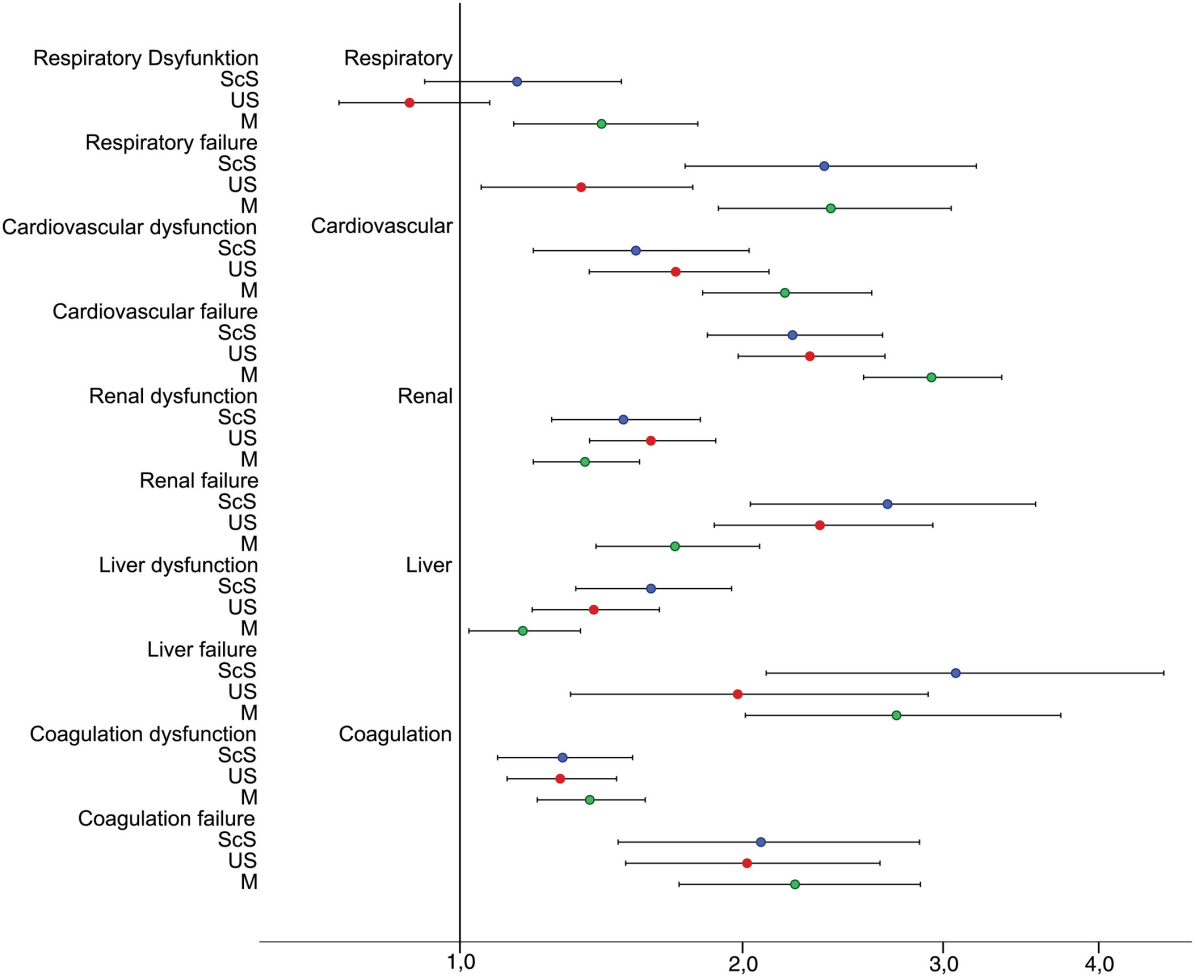
Multivariate, forward stepwise logistic regression analysis with intensive care unit mortality as the dependent factor and OD/OF at day 3. All data are adjusted to age, gender and organ dysfunction/organ failure at admission. Odds ratio is depicted in a logarithmic plotting (n = 23,795). In organ dysfunction only a significantly increased risk of death is observed in the M-patients with lung dysfunction. The highest risk of death is found in M-patients with cardiovascular or liver failure, as well as in ScS patients with liver or renal failure. In US-patients renal failure was accompanied with the highest risk of death. OR, odds ratio; CI, confidence interval; OD, organ dysfunction; OF, organ failure; CV, cardiovascular; ScS, scheduled surgery; US, unscheduled surgery; M, medical.

In the multivariate logistic regression analysis of patients with an ICU length of stay of at least 5 days, with mortality as the dependent variable, the type of admission was associated with different estimates of the risk of death in all individual organ systems except for coagulation failure (Fig 5). In the US patients respiratory failure on day three had a markedly lower odds ratio (1.38) than M (2.41) or ScS (2.37) patients. By contrast, renal failure was accompanied with a markedly higher odds ratio in surgical than medical patients (OR for ScS 2.69, US 2.35, M 1.72). For medical patients heart failure had the highest impact on mortality (OR 2.93).

## Discussion

In this retrospective analysis of registry data we found that both the prevalence of individual organ dysfunctions or failures and ICU mortality across types of admissions after three days of ICU treatment demonstrated significant variability.

Notably, in patients with heart or renal failure the consideration of the type of admission and the different risk of death of the individual organ failures appears to be an under-appreciated factor within multicenter trial design for intensive care patients.

Several organ dysfunction indices have been described, including the APACHE and SAPS scores [1, 3, 12, 13]. However, these models are limited as they predict mortality based on data collected over the first 24 hours following patient admission. As a result, these data remain static and do not include information contained within a daily organ failure score.

To date the risk of death associated with an individual organ failure, in combination with the patient’s type of admission to ICU, has been poorly investigated. Our retrospective data analysis demonstrates that patients with a medical reason for admission had a different prevalence and risk of death for individual organ dysfunctions or failures. As an organ function is a dynamic progress we were especially interested in patients with a longer period of treatment. Therefore we evaluated patients with an ICU LOS of at least five days and evaluated their organ function on ICU admission and on day three, as organ function often deteriorates immediately before death. This hypothesis is supported by an analysis of the INDEPTH database (International Integrated Database for the Evaluation of Severe Sepsis and Drotrecogin alfa activated) [8]. Notably, in the four days prior to death 80% of nonsurvivors had an increase in at least one individual organ SOFA score, while the mean changes in the individual organ SOFA scores were small. Our data are in accordance with data from the INDEPTH database and other sources showing that the most common organ failures in intensive care are lung and cardiovascular system [7, 8, 14–16]. In a retrospective analysis of data collected between 1989–99 Ostermann *et al*. investigated the impact of different types of organ failure on ICU mortality in patients with acute kidney injury [14]. The risk of death in their multivariate analysis is not directly comparable to ours as the degree of acute kidney injury was used as an independent factor, and no adjustment was made for age, gender or type of admission.

Vincent *et al*. demonstrated the influence of the type of admission on the risk of death in the SOAP study. In a multivariate logistic regression analysis of 1177 sepsis patients, using intensive care unit mortality as the independent variable, those patients with a medical reason for admission showed an OR for death of 1.4 (95% CI 1.0–1.8) [16].

### Possible Implications

Our data support the notion that the type of admission plays a role in evaluating the data of large (multi center) trials and should therefore be considered. This might hold especially true for the kind of studies that consider outcome parameters like organ failure and mortality. Based on our data, i.e. two organ failures involving the lung and cardiovascular system would results in a risk of death of 5.3 for M patients, 4.6 for ScS patients, and 3.7 for US patients. Such differences in study populations might give misleading results or lead to a failure in reproducing the results of a former study.

Consequences as different therapeutic options in the individual medical or surgical patient are difficult to suggest, as the existing study data do not differentiate between different types of admission. However, for example the awareness for a renal failure in surgical patients may be more crucial than in medical patients. A high awareness to prevent or avoid of new organ dysfunction or failure should be standard in ICU treatment.

The underlying pathophysiological reason for our finding of different risk of mortality in individual organ failure between the different types of admission is speculative. To date there is a different risk of mortality for patients with different types of admission to ICU. All scoring systems estimating ICU or hospital mortality, e.g. the APACHE or SAPS Score distinguish between the types of admission with unequal points for the different admission types. For example in the SAPS II score patients are stratified with 0 points for scheduled surgery, 6 points for medical admission and with 8 points after unscheduled surgery admission to ICU. The main difference in the patient’s history before ICU admission is the surgical trauma. This might be one reason for different pathophysiological reactions / risk of organ failure, as a surgical trauma is an additional factor influencing the innate and adaptive immunity of patients.

### Limitations

First of all this study was a retrospective investigation of a prospectively generated database of the DIVI. This should be kept in mind as far as the generalizability of the results is concerned. However the data base is a large “multicenter” database, including a high number of severely ill patients from 24 hospitals across Germany. The DIVI registry did not include data of the individual co-morbidities of the patients. For further investigations it will be crucial to distinguish also for patient’s co-morbidities. To the best of our knowledge, even in large multicenter trials including surgical and medical patient’s impact of differences in co-morbidities are not addressed. For future evaluations we have enlarged the new database structure of the DIVI registry to include missing comorbidities.

The registry is a voluntary quality database. Control of data input was completed using numerous plausibility checks, however no on-site monitoring was performed. Nevertheless, the ten years of data input, our previous experience with this data set, the large number of patients, and the highly variable degree of illness severity suggest that no relevant systemic error has occurred.

Another major limitation is that only ICU mortality was available as an outcome measure. This hospital mortality may have several limitations. For example, a patient requiring a long-lasting therapy is more likely to show a higher mortality risk. These patients might have been discharged for additional treatment to other hospitals or rehabilitation centers. As a consequence mortality would be underestimated for this group of patients.

## Conclusion

Our data demonstrate that the prevalence and the accompanying risk of death of the individual organ failure markedly differ between the different types of admission. This fact holds true especially for patients with cardiovascular or renal failure.

The type of admission and the occurrence as well as the grading of different organ dysfunctions should be considered for the approach to the individual patient. For multicenter trials including medical and surgical patients the difference made by organ dysfunction to the risk of death might introduce an imbalance affecting outcome related results.
